# Psychotic symptoms in a patient with Systemic Lupus Erythematosus: A diagnostic dilemma between lupus psychosis and steroid induced psychosis

**DOI:** 10.1016/j.amsu.2022.104843

**Published:** 2022-11-07

**Authors:** Bishnu Deep Pathak, Binit Upadhaya Regmi, Bishal Dhakal, Sushil Joshi, Nabin Simkhada, Suhail Sapkota, Sushant Joshi, Sujan Rana Thapa

**Affiliations:** a Jibjibe Primary Health Care Center, Rasuwa, Nepal; b Nepalese Army Institute of Health and Sciences, Kathmandu, Nepal; c Department of Psychiatry and Mental Health, TUTH, IOM, Maharajgunj, Nepal

**Keywords:** Systemic lupus erythematosus, Steroids, Psychiatry

## Abstract

**Introduction:**

Systemic Lupus Erythematosus (SLE) is a chronic auto-immune disorder with the involvement of multiple organ systems. It is more common in females.

**Case presentation:**

Here, we present a case of 12-year-old female, known case of SLE with lupus nephritis, presenting with neuropsychiatric symptoms. She was under steroids as well for a few weeks due to flare-up of symptoms prior to that. Due to this, there was a diagnostic dilemma between lupus psychosis and steroid induced psychosis.

**Clinical discussion:**

Approximately one third to half of the patients may have neurological involvement in Systemic Lupus Erythematosus. However, neuropsychiatric symptoms in them could be due to corticosteroids, which are frequently used in treatment. There are no definitive and easily available laboratory markers to distinguish these two aetiologies.

**Conclusions:**

Systemic Lupus Erythematosus patients, who are on steroids, with neuropsychiatric features should be assessed adequately. As there are no specific guideline and biomarkers to distinguish between these two, meticulous evaluation is necessary for appropriate management.

## Highlights

• In SLE, neuropsychiatric manifestations can be due to disease itself or drugs used to treat it.
• It is very difficult to distinguish lupus induced psychosis from steroid induced psychosis.
• There are no any definitive investigations or guidelines to differentiate between the two.
• However, risk factors like lupus nephritis, hypoalbuminemia and female sex help to separate them.
• Whatever may be the cause for the psychotic symptoms, it should be evaluated first and treated with anti-psychotics.

## 1 Introduction

Systemic lupus erythematosus (SLE) is a chronic, autoimmune inflammatory condition affecting connective tissue of multiple organ systems. In the background of susceptible gene and environmental factors, organs and cells undergo damage mediated by tissue-binding autoantibodies and immune complexes. It is nine times more common in women [1]. Studies report that approximately one-third to half of SLE patient have neurological or neuropsychiatric manifestation [2,3]. Neuropsychiatric SLE (NPSLE) refers to neurological or psychiatric syndromes seen in patients of SLE after exclusion of other causes [4].

NPSLE has broad range of manifestations including stroke, cognitive dysfunction, seizures, delirium, psychosis, peripheral neuropathies, movement disorders, cranial neuropathies, myelitis, or meningitis [2,5]. These manifestations may occur before, at the diagnosis or follow the diagnosis of SLE [6]. In 1999, case definition, diagnostic criteria, important exclusion, and method of ascertainment were developed by American College of Rheumatology (ACR) for 19 NPSLE syndromes [4]. But there is no single clinical, laboratory, neuropsychological and imaging test that can be used to differentiate NPSLE from non-NPSLE patients with similar neuropsychiatric manifestations [7]. Psychosis is seen in 1–2% of SLE patients [2,8].

Here, we present a case of 12-year-old female, previously diagnosed as SLE with lupus nephritis stage IV, now presenting with psychiatric manifestations.

## 2 Case presentation

Eight years ago, a 12-year-old female presented to our centre with rashes over face and chest along with progressive swelling of both lower limbs for one month. There were complaints of myalgia, arthralgia of both knee joints and photosensitivity. On clinical examination, the erythematous maculo-papular rashes were present over the face and chest. Facial rashes were malar in distribution with sparing of naso-labial folds. There was pallor with pitting oedema of lower limbs. The laboratory investigations at the time of presentation are shown in Table 1.

**Table 1:** Laboratory investigations at presentation.

SN	Investigations	Observed values	Reference values
1	Total leucocyte count	**3600/mm**^**3**^	4000–11,000/mm^3^
2	Neutrophils	**37%**	55–70%
3	Lymphocytes	**61%**	20–40%
4	Hemoglobin	**8.8 gm/dl**	12–16 gm/dL
5	Platelet	1,95,000/mm^3^	150 – 450 × 10^3^/mm^3^
6	Urea/creatinine	38/1.1 (mg/dL)	Urea: 12–48 mg/dL Creatinine: 0.6–1.1 mg/dL
7	Total serum bilirubin	0.8 mg/dL	0.1–1.2 mg/dL
8	Direct serum bilirubin	0.2 mg/dL	<0.3 mg/dL
9	Alkaline phosphatase	**338 IU/L**	44–147 IU/L
10	Serum Glutamic-Oxaloacetic Transaminase (SGOT)	23 IU/L	10–40 IU/L
11	C-Reactive Protein (CRP)	**Positive**	
12	Rheumatoid Arthritis (RA) factor	Negative	
13	Total serum protein	**4.7 gm/dL**	6.0–8.3 gm/dL
14	Total serum albumin	**2.9 gm/dL**	3.4–5.4 gm/dL
15	24-h urinary protein	**280.4 mg**	<100 mg
16	Lipid profile: Total cholesterol HDL cholesterol LDL cholesterol VLDL cholesterol Triglyceride	**272 mg/dL** **48 mg/dL** **195 mg/dL** 29 mg/dL 145 mg/dL	<200 mg/dL ≥60 mg/dL <110 mg/dL 2–30 mg/dL <150 mg/dL
17	Spot P/C ratio	**0.3**	<0.25

HDL: high density lipoprotein; LDL: low density lipoprotein; VLDL: very low-density lipoprotein; P/C: protein creatinine ratio.

Further investigations were done suspecting SLE and associated organ involvement. Urine routine examination showed hematuria and proteinuria. The peripheral blood showed normocytic normochromic anemia. Anti-nuclear Antibody (ANA) and anti-deoxyribonucleic antibody (Anti ds-DNA) were positive. On echocardiography, there was mild pericardial effusion and mildly dilated left ventricle. A diagnosis of Systemic Lupus Erythematosus (SLE) was made according to Systemic Lupus International Collaborating Clinic (SLICC) criteria [9].

She was then put on oral Prednisolone and oral Hydroxychloroquine. Her body weight at the time of presentation was approximately 45 kg. Considering this, the initial dose of prednisolone was adjusted as 60 mg/day (1.3 mg/kg/day) and that of Hydroxychloroquine was 200 mg/day (4 mg/kg/day). The antihypertensive drugs (Amlodipine 10 mg once daily) were also administered due to high blood pressure (her blood pressure was above 95th percentile for her age).

During the course of her hospital stay, the oedema progressed gradually throughout her whole body. Since there was proteinuria, hematuria, hypoalbuminemia and low complement levels (C3 = 10 mg/dL and C4 = 3.06 mg/dL), the treating physicians suspected renal involvement and ordered renal biopsy. The biopsy showed Lupus Nephritis stage-IV.

Following the diagnosis of Lupus Nephritis, stage-IV, she was put on induction therapy with Cyclophosphamide (1.5 mg/kg once daily) for first six months. After that, maintenance therapy was started with Azathioprine (50 mg once daily), low dose steroid (Prednisolone 30 mg/day in tapering dose) and Hydroxychloroquine (400 mg once daily). She remained symptom-free for first two years. But, afterwards, she quitted her regular medications in between on her own for two times. Due to this, she had flare-up of symptoms and was admitted to hospital.

About 20 days prior to present episode, she was admitted in medical ward with the complaints of oedema, malar rashes and decreased urine output. She was treated with parenteral steroid, oral Hydroxychloroquine and intravenous human albumin. She was discharged on oral Prednisolone (55 mg/day), oral Hydroxychloroquine (400 mg/day) and Mycophenolate mofetil (1 gm/day).

At present time, she was brought to our centre with the complaints of irrelevant talks, abnormal behaviours and elevated mood for two days. There was also complaints of progressive swelling of whole body and decreased urination for ten days. According to her parents, she had some possessive spells, and she had been behaving as a goddess. She had been trembling her body, and worshipping herself and others nearby her. There was irrelevant self-talking with disturbed sleep. The Real Time-Polymerase Chain Reaction (RT-PCR) test for COVID-19 was negative. And previously, there was no any similar history.

On physical examination, there was facial oedema with periorbital puffiness. There was bilateral pitting oedema of both upper and lower limbs. Her vitals were normal except blood pressure, which was on the higher side. Cardiovascular, respiratory and abdominal examinations were unremarkable. On mental status examination, she was restless and agitated. She was not cooperative to the examiner. Mood symptoms were predominant with delusion of grandiosity. Her speech was pressured with increased tone and volume.

The baseline investigations were sent which were normal. She was put on benzodiazepine (Clonazepam 0.25 mg twice daily) for the first three days, and then tapered slowly for the next three days, and then stopped. The second-generation antipsychotic drug (Olanzapine 5 mg) was also started, and was titrated up to 7.5 mg on the third day. Her symptoms gradually resolved over three to four days.

During the present episode, there was dilemma among the treating physicians regarding her psychiatric symptoms. There were no other evident organic and non-organic causes that could explain the symptoms. The possible aetiology was either SLE or steroid. Amidst this confusion, benzodiazepine and antipsychotic were started. At the same time, steroid was gradually tapered (10 mg per weekly and was stopped at 5 mg/day). This brought complete resolution of her psychiatric symptoms over a period of week. Later, she became cooperative and had a good sleep.

The patient was continued on Olanzapine and called for follow-up after one month. She was referred to Nephrology unit for the further needful regarding lupus nephritis.

## 3 Discussion

Neuropsychiatric manifestations in SLE may be due to the disease itself or drugs used to treat it (glucocorticoids, Chloroquine) or exacerbation of pre-morbid psychiatric condition [10,11]. These include acute confusional state, psychosis, mood symptoms, anxiety disorder, seizures, cognitive dysfunction, delirium, headache, movement disorder, etc [12,13]. Etiopathogenesis is largely attributed to increased permeability of blood brain barrier and cytokine mediated brain injuries, role of autoantibodies to neuronal antigens, phospholipid associated proteins and intracranial generation of inflammatory markers [13,14]. Prevalence of NPSLE varies from 11 to 81% in different studies and is associated with poor prognosis [2].

It is reported that steroids can cause psychosis in 4.8% of patients with SLE [15]. However, differentiation of lupus psychosis versus steroid induced psychosis is very difficult. In case of doubts, some experts suggest rapid tapering and stopping of steroids whereas others advocate increasing the dose and awaiting clinical responses [16,17]. Lupus psychosis is characterized by acute confusional state and seizure episodes whereas mood disorder is predominant in steroid induced psychosis. Likewise, the former is found to be associated with high level of antibodies to p-ribosomal proteins, increase in cerebrospinal fluid IL-6, serum anti Sm antibody and antiphospholipid antibodies [18]. Study of steroid dosage (prednisolone equivalent>40 mg/day), time interval (within one to two weeks of initiation of therapy) and duration of mental changes (improvement with tapering of steroids) can be helpful [10,19].

In our case, patient developed neuropsychiatric features after eight years of initial diagnosis of SLE. There was a history of recurrent flare-ups and lack of adherence to medication in between. The patient was on regular systemic steroid (oral prednisolone 55 mg/day) when she developed psychiatric symptoms. This brought a diagnostic dilemma amongst the physicians regarding whether it was lupus induced or secondary to corticosteroid treatment. During her in-hospital stay, the second-generation antipsychotic drug Olanzapine was started and systemic steroid was tapered. After starting olanzapine in a week time, psychotic symptoms gradually subsided and was kept in maintenance dose. She recovered gradually and was finally discharged. Considering aforementioned studies and evidences, the psychiatric symptoms in our case started a few weeks after the onset of high dose steroid therapy, were more of mood related and they relieved on tapering the dose of steroids. So, steroid induced psychosis was the close differential.

The guidelines suggest that neuropsychiatric manifestations in SLE should be first evaluated and treated as in patients without SLE, and then it should be co-related with the disease [14]. In our case also, we did the same. Our patient was put on olanzapine from very first day and then detail work-up for SLE and to rule out other possible diagnoses were done. Cases of SLE later presenting with psychiatric features on steroid use or dose adjustment are being reported in other studies [16,20,21]. But some cases initially presented with neuropsychiatric symptoms and later found to have SLE on evaluation are also reported [10,22,23]. Likewise, in one study, both conditions were found to co-exist [18].

It is seen that SLE patients with concurrent lupus nephritis and hypoalbuminemia were more prone to develop steroid induced psychosis [1]. Female gender and high dose of steroids are another risk factors [7]. In our case, the patient had lupus nephritis grade IV and at presentation, hypoalbuminemia with anasarca was also seen. So, she had a higher risk of developing neuropsychiatric features.

There were some limitations associated with this study to be mentioned. We had not been able to perform laboratory investigations, especially CSF and serum markers, suggested by different past studies due to lack of such facilities in our centre. There was no precise definition available about patient and her steroid dependency. Moreover, this case was encountered during the COVID-19 pandemic which hindered our adequate evaluation and management process. However, we had followed up this case since the initial diagnosis and every possible detail of the patient has been properly assessed. We tried our best to execute appropriate management by close coordination between general physicians, neuropsychiatrists and nephrologists. This is our strength associated with the case.

## 4 Conclusion

SLE patient presenting with neuropsychiatric features should be assessed properly. Primary psychiatric disorder and other differential diagnoses should be ruled out. There is a need of appropriate diagnostic guideline to distinguish neuro-lupus from steroid induced psychosis. Biological markers described so far also do not have definitive evidence and they are not available easily. Therefore, easily accessible investigations are necessary. Since female sex, lupus nephritis and hypoalbuminemia have higher susceptibility of developing steroid induced psychosis, these cases should be given special focus. Liaison with neuropsychiatrists is recommended for better outcome.

## Author agreement statement

We the undersigned declare that this manuscript is original, has not been published before and is not currently being considered for publication elsewhere. We confirm that the manuscript has been read and approved by all named authors and that there are no other persons who satisfied the criteria for authorship but are not listed. We further confirm that the order of authors listed in the manuscript has been approved by all of us.

We understand that the Corresponding Author is the sole contact for the Editorial process. He/she is responsible for communicating with the other authors about progress, submissions of revisions and final approval of proofs.

## Ethical approval

This is a case report, therefore, it did not require ethical approval from ethics committee.

## Funding

The study did not receive any grant from funding agencies in the public, commercial or not-for-profit sectors.

## Author contribution

1. Bishnu Deep Pathak.

2. Binit Upadhaya Regmi.

3. Bishal Dhakal.

4. Sushil Joshi.

5. Nabin Simkhada.

6. Suhail sapkota.

7. Sushant Joshi.

8. Sujan Rana Thapa.

## Consent

Written informed consent was obtained from the patient for publication of this case report and accompanying images. A copy of the written consent is available for review by the editor-in-chief of this journal on request.

## Trail registry number

Not applicable.

## Guarantor

Bishal Dhakal, Nepalese Army Institute of Health and Sciences, 44600 Kathmandu, Nepal. Email: swarnimdhakal@gmail.com, Phone: +977 9846491651.

## Declaration of competing interest

The authors report no conflicts of interest.
